# The role of structural and interpersonal violence in the lives of women: a conceptual shift in prevention of gender-based violence

**DOI:** 10.1186/s12905-015-0247-5

**Published:** 2015-10-26

**Authors:** Stephanie Rose Montesanti

**Affiliations:** School of Public Health, University of Alberta, Edmonton, AB Canada

Explanations for violence against women (VAW) have developed in a wide variety of disciplines including, sociology, psychology, social work and public health. Theories of VAW range from individual and relationship level explanations to socio-cultural and political explanations for why violent acts towards women are committed. Feminist scholars, for instance, focus their attention to male-dominated social structures and socialization practices that teach men and women gender-specific roles that can influence violence and abuse against women [1]. One of the most common forms of violence against women is interpersonal violence (IPV). IPV refers to everyday violence such as sexual and physical assault that occurs between family members, intimates, or acquaintances.

The UN Declaration was the first international statement that defined violence against women within a broader gender-based framework and identified the family, the community and the state as major sites of gender-based violence. The statement was rooted in feminist analysis of social inequality. According to the UN Declaration, violence against women involves:

- Any act of gender-based violence that results in, or is likely to result in physical, sexual or psychological harm or suffering to women, including threats of such acts, coercion or arbitrary deprivation of liberty, whether occurring in public or in private life. (p.1)

*Gender-based violence (GBV)* can include domestic violence, sexual harassment, sexual violence and rape. GBV is a deliberately broad term in order to recognize the gendered elements in nearly all forms of violence against women and girls, whether it is perpetrated through sexual violence or through other means. The use of the term ‘gender-based violence’ provided a new context in which to examine and understand the phenomenon of violence against women. It shifted the focus from women as victims of violence to gender and the unequal power relationships between women and men that are created and maintained through gender stereotypes. A gender perspective on violence against women addresses the similarities and differences in the violence experienced by women and men in relation to vulnerabilities, violations and consequences.

In response to this declaration, various efforts have been made to respond to reduce and eliminate this violence experienced by women. Significant attention has been paid in the Northern hemisphere and high income countries such as Canada and the U.S. to the provision of social services to victims of GBV, such as strengthening and maintaining women’s safety and their involvement in social, political and economic activities. Changes have also been made to justice sector responses, and to treatment for perpetrators of GBV.

Interventions in low and middle-income countries have focused on primary prevention strategies to reduce the prevalence and incidence of violence against women and girls. These prevention programs use a wide range of approaches, including group training, social communication, community mobilization, and livelihood strategies. Microfinance and cash transfer programs in countries such as South Africa, Kenya and Ecuador have reported reductions in the rates of IPV [2, 3]. Community mobilization programs in Uganda and Sub-Saharan Africa that aim to reduce violence at the population-level through changes in public discourse, practices, and norms for gender and violence, demonstrated not only reductions in physical and sexual partner abuse, but also reduced incidence of HIV/AIDS [4, 5].

These responses, however, have largely turned to understandings of GBV that place the causes, consequences and costs at an individual level [6]. With the launch of the World Health Organization (WHO) Multi-Country Study on Women’s Health and Domestic Violence in 2005, the number of IPV prevalence studies increased. This research primarily from the health and medical fields has largely focused on individual or relationship level factors to the exclusion of factors operating at a broader societal level [7, 8]. Prevalence studies from the around the world have shown that IPV has a number of health consequences for that include, injury, chronic pain, sexually-transmitted diseases, depression, post-traumatic stress disorder, to name a few [9–15]. Though this research has contributed to an understanding of the prevalence [16], consequences [9, 17], and costs [18, 19] associated with IPV against women; its focus has been on individual behaviors and health outcomes, ignoring how patterns of violence are connected to social systems and social institutions. Any analysis of violence “must recognize the primacy of culturally constructed messages about the proper roles and behavior of men and women and the power disadvantaged women bring to relationships by virtue of their lack of access to resources.”[14]

In this thematic series, the authors provide an in-depth analysis of how social systems and institutions influence interpersonal violence that disproportionately harms women. *Structural violence* has been defined as the social arrangements that put individuals and populations in harm’s way…the arrangements are structural because they are embedded in the social, political and economic organization of our social world; they are violent because they cause injury to people (typically, not those responsible for perpetuating such inequalities) [20]. Structural violence includes “a host of offensives against human dignity: extreme and relative poverty, social inequalities ranging from racism to gender inequality, and the more perverse forms of violence that are uncontestably human rights abuses” [21]. In adopting a structural violence approach to understand GBV in a variety of contexts and events, our analysis underscores the importance of historical and social contexts that influence IPV towards women.

Structural violence is marked by unequal access to the determinants of health (e.g., housing, good quality health care, unemployment, education), which then creates conditions where interpersonal violence can occur and shape *gendered forms of violence* that place women in vulnerable positions. Gender is inescapably embedded in social systems and institutions. For instance, Parikh (2012) illustrates how a macro-level structural intervention (increase in the age of consent law at national and local levels) intended to address gendered HIV risk in Uganda has the unintended consequence of reinforcing gender–based social hierarchies [22]. Despite the stated aim of protecting young women, the law reinstates patriarchal privilege and the regulation of adolescent female sexuality. Moreover, research pertaining to gender tends to regard the categories of “men” and “women” as distinct categories, not just in their biological make-up but also in their gender-specific role socialization.

This thematic series originated through the Canadian Institute for Health Research (CIHR) Institute of Gender Health (IGH) Roundtable on Violence, Gender and Health on January 28–29, 2010, in Ottawa, to discuss the current state of research on gendered violence and health. Participants at the meeting identified two research priorities: (1) the need to conduct contextualized research on how social systems and institutions perpetuate and reproduce gender-based violence against women and impact various dimensions of health; and (2) the need to examine individual-level characteristics and population-level influences on gender-based violence. Initial discussions at this meeting brought together a team of researchers to develop a comparative program of research to advance our understanding of structural and systemic violence in gender-based violence and women’s health inequities in the Canadian context, which includes an analysis of appropriate interventions to prevent future occurrences of violence against women.

This thematic series will advance scholarly knowledge on the two research priorities noted above, and underscore new ways of thinking about structural and interpersonal violence, and how they are related and manifest in the lives of women. The series will include a collection of empirical and theoretical papers that engage a variety of methodological approaches and signal the growing challenges in thinking through, and responding to, gender-based violence. It will demonstrate the need for researchers to continue interrogating categories in their analyses, including gender and sex. Also urging researchers and policy makers to think beyond an individualized focus on the causes, consequences and costs of violence and abuse, to the implementation of interventions, in the form of health and social services and policies, that consider the contexts in which people’s lives are experienced.
